# The Efficacy of Mesenchymal Stem Cell Transplantation in Caustic Esophagus Injury: An Experimental Study

**DOI:** 10.1155/2014/939674

**Published:** 2014-05-04

**Authors:** Murat Kantarcioglu, Bahadir Caliskan, Hakan Demirci, Ozgur Karacalioglu, Murat Kekilli, Zulfikar Polat, Armagan Gunal, Melih Akinci, Cagri Uysal, Sami Eksert, Hasan Gurel, Gurkan Celebi, Ferit Avcu, Ali Ugur Ural, Sait Bagci

**Affiliations:** ^1^Department of Gastroenterology, Gulhane Military Medical Academy, Etlik, 06108 Ankara, Turkey; ^2^Department of Pediatric Surgery, Gulhane Military Medical Academy, Etlik, 06108 Ankara, Turkey; ^3^Department of Nuclear Medicine, Gulhane Military Medical Academy, Etlik, 06108 Ankara, Turkey; ^4^Department of Gastroenterology, Hitit University, 19030 Corum, Turkey; ^5^Department of Pathology, Gulhane Military Medical Academy, Etlik, 06108 Ankara, Turkey; ^6^Department of General Surgery, Ankara Diskapi Training and Research Hospital, 06110 Ankara, Turkey; ^7^Department of Plastic and Reconstructive Surgery, Faculty of Medicine, Baskent University, 06100 Ankara, Turkey; ^8^Department of Hematology, Gulhane Military Medical Academy, Etlik, 06108 Ankara, Turkey; ^9^Department of Hematology, Bayindir Hospital, 06250 Ankara, Turkey

## Abstract

*Introduction.* Ingestion of corrosive substances may lead to stricture formation in esophagus as a late complication. Full thickness injury seems to exterminate tissue stem cells of esophagus. Mesenchymal stem cells (MSCs) can differentiate into specific cell lineages and have the capacity of homing in sites of injury. *Aim and Methods.* We aimed to investigate the efficacy of MSC transplantation, on prevention of esophageal damage and stricture formation after caustic esophagus injury in rats. 54 rats were allocated into four groups; 4 rats were sacrificed for MSC production. Group 1, untreated controls (*n*: 10). Group 2, membrane labeled MSCs-treated rats (*n*: 20). Group 3, biodistribution of fluorodeoxyglucose labeled MSCs via positron emission tomography (PET) imaging (*n*: 10). Group 4, sham operated (*n*: 10). Standard caustic esophageal burns were created and MSCs were transplanted 24 hours after. All rats were sacrificed at the 21st days. *Results.* PET scan images revealed the homing behavior of MSCs to the injury site. The histopathology damage score was not significantly different from controls. However, we demonstrated Dil labeled epithelial and muscle cells which were originating from transplanted MSCs. *Conclusion.* MSC transplantation after caustic esophageal injury may be a helpful treatment modality; however, probably repeated infusions are needed.

## 1. Introduction

Mostly in developing countries, caustic ingestion continues to be a significant medical problem in different age groups and is a leading cause of morbidity and mortality in the pediatric population [1]. Ingestion occurs as an intentional exposure in adults, while children's exposure is accidental. The basic pathophysiologic reaction of tissue subjected to caustic burn is the deposition, synthesis, and remodeling of extracellular matrix, and following full thickness injuries to the wall of esophagus, the normal esophageal tissue is replaced by connective tissue [2, 3].

Today, the aim of therapy in corrosive esophageal burn injuries is to prevent development of fibrosis, stricture formation, and perforation [4]. Several types of nonsurgical management such as steroid injections, antibiotics, esophageal dilatation, stent applications, and surgical treatment options are used for treatment [2, 4–6]; but current types of treatment are quiet ineffective for severe corrosive burns. In experimental models, some agents such as vitamins E and C, heparin, mitomycin, penicillamine, caffeic acid, epidermal growth factor, interferon g, sphingosylphosphorylcholine, phenethyl ester, progesterone, and estradiol are found to be effective in different degrees [7–13]. However, they have not been applied as standard treatment protocols for corrosive esophageal burn injuries at present.

Mesenchymal stem cells (MSCs) constitute an alternative source of pluripotent stem cells. They have the capacity to differentiate into cells of mesodermal lineage, and also have a much broader differentiation potential. Cells with similar properties exist in a variety of other tissues, including adipose tissue, peripheral and umbilical cord blood, placenta, amniotic fluid, fetal tissues, synovial membrane, and deciduous teeth, but bone marrow MSCs are the best characterized ever [14–16]. MSCs also have the capacity to home in sites of injury [17]. Recent studies have shown that bone marrow-derived MSCs might play an important role in the repair processes of injured tissues [18, 19]. Okamoto et al. reported that bone marrow derived cells can repopulate esophagus epithelia in humans [20]. They also have shown that bone marrow derived cells are a potential source from which to repopulate esophagus epithelia especially during episodes of inflammation and regeneration, raising the possibility that clinical therapies to regenerate human esophagus epithelia with bone marrow cells may be possible [20]. Ringdén et al. reported that MSC transplantation plays a role in healing tissue toxicity in patients who had complications after allogeneic hematopoietic stem cell transplantation [21]. In addition, several groups have demonstrated the ability of MSCs to secrete angiogenic factors, such as vascular endothelial growth factor, hepatocyte growth factor, and interleukin-6 both in vitro [22, 23] and in vivo [24].

There has been no evidence about any effect of MSCs on improving any form of esophageal injury. The aim of this experimental study was to investigate the efficacy of allogeneic MSCs, which intravenously transplanted into a caustic esophagitis rat model. In this context we aimed to assess their homing behaviour towards the esophagus injury site and improve capacity on mucosal healing and reversing stricture formation.

## 2. Materials and Methods

This study was approved by the Experimental Ethical Committee of the Gulhane Military Medical Academy, Ankara, Turkey.

### 2.1. Study Groups

In order to have 90% power with 5% type I error level to detect a minimum clinically significant difference of 50%, 20 rats for treatment group and 10 rats for each left group (sham, control etc.) had to be recruited to the study.

The study was performed using 54 albino, male, 2-month-old Sprague-Dawley (SD) rats weighing between 250 and 300 g each. All the rats were obtained from our animal research center. Rats were kept in experimental animal production cages which were closed with plastic on bottom and sides and top with wire fence. Room temperature was maintained at 22°C. In order to simulate circadian rhythm, 12 h light and dark periods were carried out. The rats were fed by pellet type fabrication feed that is produced specially for experimental animals. Four rats were sacrificed and their tibias and femurs were excised. Their marrows were cultured to obtain MSCs. The remaining 50 animals were randomly allocated into 4 groups. Group 1 (control group), in which caustic esophageal burns were created but left without treatment (10 rats). Group 2 (MSCs-treated group), in which animals with caustic esophageal burns were transplanted, and membrane labelled allogeneic bone marrow derived 1 × 10^6^ mesenchymal stem cells each one day after injury. They were sacrificed at 21st day and histopathologic analysis was performed to esophagus specimens (20 rats). Group 3 (MSCs tracked via PET imaging) in which stem cells were labeled with fluoro-D-glucose (FDG), and after transplantation, MSCs were tracked for 4 hours via positron emission tomography in order to observe their early homing behaviour (10 rats). Group 4 (sham-operated group), in which laparotomy was performed, esophagus was uninjured and untreated (10 rats).

### 2.2. Experimental Model

After 12 hours of fasting, each rat was anesthetized intraperitoneally with xylazine hydrochloride (15 mg/kg) and ketamine hydrochloride (100 mg/kg). A standardized esophageal caustic burn injury was produced using the method described by Gehanno and Guedon [25]. Applying sterile surgical techniques, a midline laparotomy was made and 2 cm of abdominal esophageal segment was isolated and tied with 2/0 silk sutures distally and proximally. A 24-F cannula was placed into the isolated segment through a gastric puncture. The esophageal injury was created by instilling 20% NaOH solution for 3 minutes until slight translucency of the esophageal wall and branching of the vessels were noted, and then the solution was aspirated. Subsequently, distilled water was used to irrigate the injured segment for a 60-second period. In the sham operated group, distal esophageal segments were instilled with 0.09% NaCl solution only. The laparotomy incision was closed and 10 mL of saline was administered subcutaneously in each animal. Rats were not allowed to feed for the next 24 h. All animals were kept in identical cages that provided food and water during the study period. On the 21st postoperative day, the rats were decapitated and distal 1.5 cm esophageal segments were harvested for histopathologic investigations. Specimens were placed in 10% buffered formaldehyde solution.

### 2.3. Bone Marrow (BM) Preparation and BM-Derived Rat MSC Generation

Briefly, 4 SD rats were sacrificed by decapitation and Bone Marrow (BM) was flushed with L-DMEM (Gibco Lab, Grand Island, NY) using a 23-gauge needle from femurs and tibias. The BM cells were then pelleted by centrifugation at 1000 rpm for 15 min. The BM cells were gently resuspended using an 18-gauge needle and filtered through a sterile nylon mesh. The viability was consistently >95% as determined by trypan blue exclusion. For the MSC generation, BM cells were plated in 25 cm^2^ polystyrene flasks in L-DMEM supplemented with 10% fetal bovine serum (FBS) in 37°C with 5% CO^2^ conditions (Gibco Lab, Grand Island, NY). Cells were allowed to adhere for 72 h followed by the removal of nonadherent cells and media changed every 3 to 4 days. Adherent cells were detached using trypsin-EDTA solution-B (EDTA 0.05%, Trypsin 0.25%, with Phenol Red, Biol. Ind., Israel) at 37°C for 10 min, and MSCs were expanded 3-4 times to achieve the desired cell numbers for use in vitro and in vivo experiments. MSCs were resuspended in DMEM and diluted to a final concentration of 1 × 10^6^ cells/1 mL.

### 2.4. Dil Labeling of MSCs

In order to reveal the faith of the transplanted MSCs in injured tissue, for tracking procedure, the cells were labeled by 1,1-dioctadecyl-3,3,3,3-tetramethylindocarbocyanine (DiI); Vybrant CM-DiI cell-labeling solution (Molecular Probes Inc.). MSCs were suspended in DMEM with 1 × 10^6^ cells in 1 mL and labeled with fluorescent DiI according to the manufacturer's recommendations [26]. Briefly, 5 *μ*L of cell labeling solution was added per mL of cell suspension. After mixing well by gentle pipetting, cells were incubated for 20 minutes at 37°. Then the labeled suspension tubes were centrifuged at 1500 rpm for 5 minutes and supernatant was removed. Following labeling, the MSCs were incubated with beta-TCP in a culture medium. Positive DiI staining was confirmed, and for each recipient rat, 1 × 10^6^ cells in 1 mL serum were injected via tail vein of the recipient rat.

### 2.5. PET Imaging

Stem cells were labeled with fluoro-D-glucose (FDG) following protocols similar to those described previously [27]. The activities of FDG, administered to the cell samples, were measured with a standard dose calibrator (Capintec CRC 10, Capintec Inc., Ramsey, NJ, USA). Cells were detached under mild conditions in 0.05% trypsin/0.02% EDTA (Biochrom, Berlin, Germany) for 5 minutes. The cell suspension was centrifuged at 800 g for 5 minutes and supernatant was removed. The cell pellet was resuspended in phosphate-buffered saline (PBS), and recentrifuged at 800 g for 5 minutes. For cell counting, the washed pellet was resuspended in 1 mL of PBS; the cell concentration was measured by microscopic examination of 10 *μ*L portions of the suspension. For labelling, 1 × 10^6^ cells were mixed with 2-deoxy-2-(18F) fluoro-D-glucose (FDG) at dose of 0.5 mCi/0.5 mL in plastic gamma tube and incubated for 45 minutes at 37°C in the benmari. At the end of the incubation period, the FDG-containing medium was removed and the MSCs were washed twice with PBS and they were resuspended with 0.3 mL saline.

The animals (which had been kept fasting for at least 4 h) were anesthetized with ketamine (15 mg/kg), and 37–47 MBq (1.0–1.3 mCi) and FDG labeled stem cells (1 × 10^6^ cells labeled with ~16 × 10^6^ MBq FDG/300 *μ*L) were injected via intravenous tail vein. After 60 min of distribution, the rats underwent a whole body-imaging acquisition including 6 min for the 3-dimensional-mode emission scan and less than 10 seconds for the transmission scan in PET CT camera (GE Discovery 690, WI, USA). The images were reconstructed with optimized parameters. The FDG PET CT imaging of the animals was repeated to demonstrate the gastroesophageal junction 1 hour after the oral contrast (2 mL barium sulfate suspension) administration. The animals were sacrificed by decapitation method; gastroesophageal junctions were resected and reimaged by PET scanner.

### 2.6. Histopathologic Evaluation

All samples were detected by a blind gastrointestinal system pathologist. After 10% formalin fixation, esophageal resection samples were embedded paraffin blocks crossly. 5 *μ*m thick sections were taken and then, stained with hematoxylin and eosin (HE) for general histologic evaluation and with Masson's trichrome for detecting collagen deposition to evaluate fibrosis. Firstly, with an automatized microscope-camera system (OlympusMicro DP-BSW Ver. 3.3, Olympus Co., Japan), the thickness of the esophageal wall (WT) and lumen diameter (LD) was measured, and the stenosis index (SI) was calculated to evaluate the degree of the stricture (SI = WT/LD). Secondly, tissue damage was scored on a scale based on 3 different categories described by Guven et al. (Table 1) [28].

The specimens were also examined by Nikon eclipse 80i microscope through Y-2EC filter (Wavelength; ex: 540–580 DM 595 BA 600–660) in order to visualize the differentiated DiI labeled MCSs.

### 2.7. Statistical Analysis

All statistical analyses were carried out using SPSS 15 (SPSS Inc., Chicago, IL, USA) statistical software for Windows. All data were presented as mean ± standard deviation. Differences in measured parameters among the three groups were analyzed by the Kruskal-Wallis test. Dual comparisons between groups that present significant values were evaluated with the Mann-Whitney *U* test. Differences were considered as significant when the *P* value was less than 0.05.

## 3. Results

All rats which stayed alive for 21 days were evaluated. 44 rats survived throughout the study (6 rats died; 4 from MSC treated group and 2 from control group). All animals in the sham-operated group survived during the study while two control and 4 MSCs treated rats died on different days during follow-up, after creating esophageal burns.

### 3.1. PET Imaging: FDG Labeled MSC Tracking

**Figure 1:**
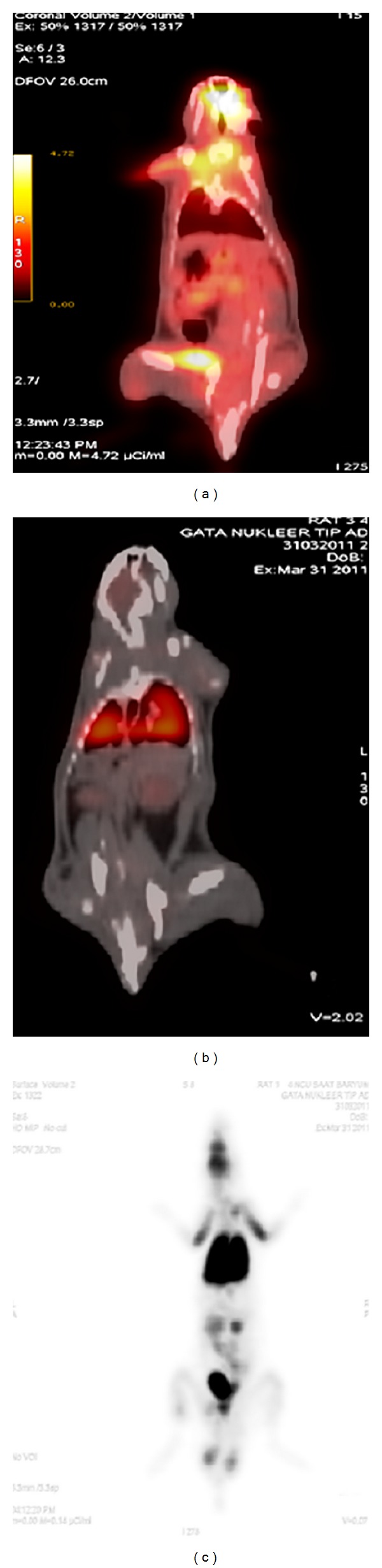
PET/CT image demonstrated the altered biodistribution between FDG (a) and the labeled stem cells with FDG (b). Prominent accumulation of the labeled stem cells in the lung was demonstrated by PET/CT (B) and PET image (c).

**Figure 2:**
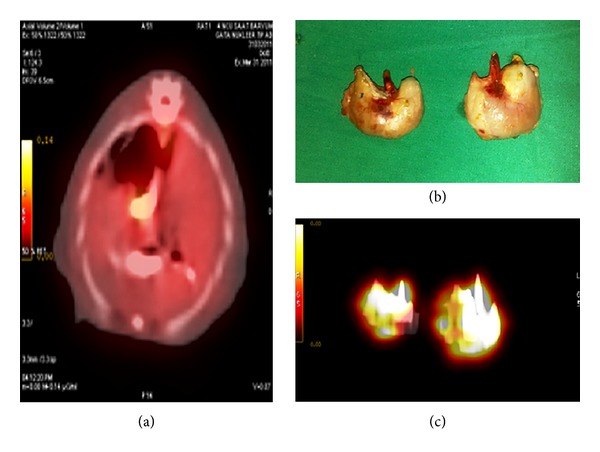
PET/CT image demonstrated the presence of the activity that can be interpreted as to be the activity of the labeled stem cells in the lower portion of the esophagus containing barium sulphate (a). Esophagogastric junction was resected (b) and imaged (c) by PET/CT to delineate the prominent activity in the lower portion of the injured esophagus.

When the FDG alone was injected into the tail vein of the rats (*n*: 2), prominent FDG uptake was noticed in the central nervous system, muscles of extremities, in liver, and spleen. There was also prominent activity accumulation in the urinary system due to physiological excretion of the FDG (Figure 1(a)). On the other hand, 1 hour after the injection of the labeled stem cells into the tail vein of the rats (*n*: 8), prominent activity caused by labeled stem cells in the lung was detected (Figures 1(b) and 1(c)). Actually this finding can be expected when labeled cells with radiopharmaceuticals such as white blood cells labeled with Tc-99 m HMPAO were reinjected intravenously; homing of the labeled cells in the lung is a usual finding on the scan acquired at the first hour [29]. There was also prominent activity in the lower portion of the injured esophagus (Figures 2(a)–2(c)). Little activity in the urinary system and in the central nervous system was also noticed due to unbound FDG. Altered biodistribution between the labeled stem cells with FDG and the FDG alone revealed the success of the labeling process.

### 3.2. Morphologic and Histopathologic Evaluation

At the end of the study, the esophagus specimens of the sham-operated group rats were macroscopically normal and there was no adhesion (*n*: 10). In contrast, those of MSCs treated (*n*: 16) and control animals' (*n*: 10) showed considerable or complete esophageal obstruction, severe adhesion (possibly due to perforation), and residual chow in the esophageal lumen. There was no significant difference between SI values of MSC treated and control groups. This finding was supported with histopathologic evaluation. The SI in the treatment and control group was significantly higher than sham-operated groups (*P* < 0.01).

### 3.3. Histologic Tracking of DiI Labeled MSCs in Injury Site

**Figure 3:**
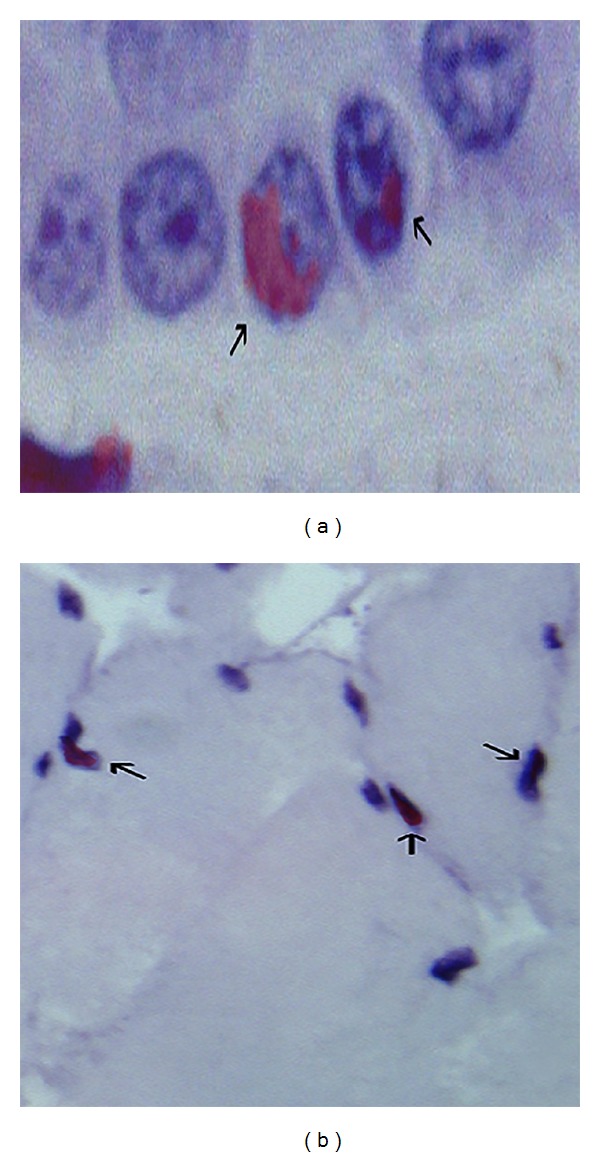
Epithelial differentiation of Dil labeled MSCs (a). Muscle cell differentiation of transplanted bone marrow derived Dil labeled MSCs (b).

The rats of Group 2 (*n*: 16) with caustic esophagitis which received Dil labeled MSCs treatment were sacrificed at the 21st day of caustic injury. The specimens were also examined by Nikon eclipse 80i microscope through Y-2EC filter (Wavelength; ex: 540–580 DM 595 BA 600–660). We noticed some esophagus epithelium (Figure 3(a)) and muscle cells (Figure 3(b)) stained positively for Dil which were originated from BM derived MSCs. The homing behaviour of MSCs that we have demonstrated by PET imaging resulted in differentiation to epithelium and muscle cells. However, the number of differentiated cells was far apart from restoring the original architecture.

## 4. Discussion

Ingestion of caustic materials is a major health problem with a high degree of morbidity [1]. Extensive damage to the esophagus can cause serious problems such as fibrosis and stricture formation [3]. Like many organs, esophagus was shown to have stromal proliferative “stem” cells by some illuminative studies carried out, regarding murine and human samples. Those mentioned cells seem to lie mostly in basement membrane of the esophagus [30]. The devastating effects of especially alkaline agents on esophagus wall by causing full thickness injury are well known. The destruction of esophageal stem cells during caustic injury and loss of regeneration capacity of the organ could easily be asserted for fibrotic healing process instead of renewal, after such a demolition. There is no valid algorithm accepted for the treatment of corrosive esophagitis [2, 3]. Esophageal stricture formation after caustic injury occurs at approximately 21 days and is completed from 28 to 42 days [31]. It is now widely accepted that the acute inflammatory response dominates the first week after injury, whereas the second week is the setting for fibroblastic proliferation and collagen formation, which represent the pathophysiological pathway of stricture formation [32, 33]. A successful management of corrosive injury involves prompt recognition and early treatment.

MSCs are a type of mesodermal-derived multipotent stem cells, found mainly in connective tissues, organs, and especially abundant in the bone marrow. Because of their multidirectional differentiation and self-replicating feature, bone marrow derived MSCs are considered the most potent for therapeutic application. Under specific induction conditions, bone marrow derived MSCs can differentiate into multiple types of tissues, such as bone, fat, cartilage, muscle, tendon, ligament, neural, liver, myocardium, and endothelial cells in vivo or in vitro [15, 16, 34]. The multipotency of bone marrow derived MSCs can be well conserved even after multiple processes [35–38]. In recent years, it has been reported that allogeneic MSCs appear to suppress graft versus host disease (GvHD) and Crohn's disease as well as induce regenerative phenomena in the case of stroke, infarct, spinal cord injury, meniscus regeneration, tendinitis, acute renal failure, and heart disease in both human and animal models of disease [39].

Up to date, there has been no research related to the effect of MSCs transplantation on any form of esophageal disease. In this experimental study, we examined whether the MSCs transplantation treatment modality has a beneficial effect on caustic esophageal injury.

Stem cells which are more prone to differentiate to specialized cell types might have been used for this experimental procedure like pluripotent stromal cells, isolated from different organs. For example, Musina et al. isolated MSCs from menstrual bloods of volunteer women [40]. In the same context, Hida et al. demonstrated that those same cells are very capable of differentiation to cardiac precursor-like cells [41]. Same sort of cells originating from endometrium, with their high differentiation capacity to muscle cells, seems like a good candidate to restore esophagus wall and their efficacy may be sought in our experimental treatment model. Also another alternative for cell source may be the stem cells derived from human placenta and fetal membranes where they are obtained without any invasive procedures and in vitro expansion potential is significantly higher than BM derived MSCs [42]. However we have chosen BM derived MSCs with their well-known features.

Throughout the experimental process, we infused MSCs only once. However the pathology investigation results of esophagus specimens were far away from restoring the original esophagus architecture. Here we think a reasonable approach may be performing multiple infusions of MSCs in order to achieve a sustained regeneration. Transplantation of MSCs via direct injection into injury site seemed also a feasible way for this study. However, after 20% NaOH injury, affected esophagus wall sites were very fragile and prone to perforation. Therefore direct implantation of MSCs may have resulted in perforation and higher animal mortality rates.

Study results have shown that single dose MSCs transplantation did not improve the esophageal injury in rats; nevertheless, animal use to predict human response to treatment modalities is apparently a contentious issue [43]. Since rodent esophagus does not resemble the human's accurately, human tissue responses to MSC transplantation may be more satisfactory. On the other hand, this experimental study demonstrated intravenously injected MSCs' first step in circulation and their subsequent homing to injured tissues in the esophagus. Histopathology results confirmed both the homing behaviour and differentiation properties of transplanted MSCs. These results provide evidence for clinical application as a treatment modality for esophageal diseases in humans. Nevertheless, time course and dose dependent studies with different types of stem cells are necessary to determine the proper timing and dosage after the injury and effects on other organs and systems.

## Figures and Tables

**Table 1:** Criteria for histopathologic evaluation.

Criteria	Score
Increase in submucosal collagen	
None	0
Mild (submucosal collagen at least twice the thickness of the muscularis mucosa)	1
Marked (submucosal collagen more than twice the thickness of the muscularis mucosa)	2
Damage to muscularis mucosa	
None	0
Present	1
Damage and collagen deposition in the muscularis propria	
None	0
Mild (collagen deposition around the smooth muscle fibers)	1
Marked (same as mild, with collagen deposition replacing some of the fibers)	2
